# The Value of a Correct Articulation in Prosthodontia

**Published:** 1904-02-15

**Authors:** Harvey M. Kirk

**Affiliations:** Los Angeles, Cal.


### THE VALUE OF A CORRECT ARTICULATION IN PROSTHODONTIA.

BY HARVEY M. KIRK, D.D.S., LOS ANGELES, CAL.

Faultless articulation, in connection with artificial dentures, is of superlative importance. If proper articulation be a feature to be observed in a fixed structure, such as a bridge or a crown, of how much greater importance is it in the case of an unfixed, or movable artificial denture, when it plays such an important part in the retention of the denture!

On account of the limited knowledge of many dentists, in connection with this subject, it is not usually conceded, nor even understood, how the perfect articulation of a denture of any kind is only second in importance to the impression itself.

But knowledge of the subject, coupled with experience, will demonstrate to an observing one that more artificial dentures are failures on account of incorrect articulation than from imperfect impressions or models. To become acquainted with the rules of normal dental arrangement and the laws governing articulation is to become fascinated enough to make one an enthusiast upon the subject.

This statement is made guardedly, and after watching its application for several years in one of the leading dental schools of the country.

Its study elevates one to a higher and larger dental realm than before, and places a more intelligent and interesting interpretation upon dental prosthesis; it makes one to see that the construction of an artificial denture, *as it should be,* is more than a mechanical jumbling together of imitation teeth and vulcanized rubber, at the instance of a hinge-like articulator; it gives one a keener enjoyment in construction and manipulation, and, the other principles being well observed, places success more uniformly within one's grasp.

Such, at least, is the experience of those who have "entered in." The late Dr. Bonwill was the one who blazed the way into this new country. It was he who first demonstrated practically the wonderful mechanical and geometrical arrangement of the jaws and teeth.

Happily, others have followed in his wake, and have had disclosed to them many more remarkable features in this connection.

It is only necessary, to mount properly upon an ANATOMICAL articulator the models of a normally-developed mouth with both natural dentures perfect, to perceive, at almost a glance, these fundamental principles, and especially so, if one have a teacher.

In this day, nearly every dentist has heard, in a more or less remote way, of the Bonwill method of articulation. Nearly every recent graduate of dentistry can explain, to some degree, its meaning; but how few are there, who are actually able to construct a full upper and lower denture upon the principles therein set forth! Yet it can be done; and in order to secure the highest success in the realm of Prosthodontia, it should be one of the accomplishments of the progressive dentist.

That it is *not* is due, primarily, to the lack of teaching, and secondly, to a lack of industry on the part of the student to find the "key" after which it becomes an easy and pleasant operation.

The Bonwill, or normal, articulation only contemplates what nature itself designed, and there is little probability of improving upon nature.

It will be observed, in the properly-articulated models of a perfect natural denture, that there are two fundamental *conditions*, or relations, of the jaws and the teeth; to-wit:

### OCCLUSION AND ARTICULATION.

Occlusion is what is wanted when the "bite" is taken, and is of itself but one *movement* or *relation* of the lower jaw to the upper, and that is, the up-and-down, hinge-like movement, terminating when the jaws are at rest, the teeth all antagonizing each other properly, and the condyloid processes secure in the glenoid fossæ of the temporal bones.

*Articulation* contemplates not only occlusion but *all* the various movements of which the jaws are capable.

Upon the mounted models (and arrangement and articulation *must* be studied in this way), it will be seen upon moving the arches of the anatomical articulator, in any direction, forward, backward or laterally, three points of the teeth will be in contact—at the incisor, and each molar region. (If the models should seem not to give this test, it will, in all probability, be due to inaccurate mounting.)

If this be true regarding the natural organs of mastication, which are firmly fixed, it is only reasonable to conclude that artificial dentures, so articulated, would not only be following out nature's design, but that the tipping of the plates, in the various movements of articulation would be largely prevented.

Not only that, but the masticatory function would be more easily performed; *especially* if the cusps of the bicuspids and molars be lengthened and sharpened, by the grinding and deepening of their sulci.

As to the possibilities of thus articulating artificial teeth, it may be stated that the limitations are by no means so great as is usually supposed; for the system may be used advantageously, not only in connection with full upper and lower dentures, but in full uppers, full upper and partial lowers, and many partials, to a greater or less extent.

Of course, perfectly-articulated artificial dentures are worthless, unless the models of the jaws upon which the artificial dentures were made be articulated, so as to assume the same relation to each other and to the articulating joint, upon the anatomical articulator, as found in the natural jaws of the patient.

Hence, getting the proper occlusion, or bite, is of the utmost importance in this connection; and the models can only be mounted accurately and correctly upon the anatomical articulator by means of the face-bow.

But this leads to a different branch of the subject, not intended to be a part of this paper.

### ARRANGEMENT.

The subject of the arrangement of the teeth is really preliminary to their articulation. There are many beautiful and fascinating points in this connection.

All articles written on the subject of the Bonwill articulation give the main features, including the triangles of the upper and lower jaws, needing only time and industry on the part of the learner to comprehend the subject. One very pretty and valuable method of locating the teeth, upon an *entirely edentulous* pair of jaws, is as follows:

Have in hand a plaster model of a perfectly-normal, upper, natural denture, teeth all intact.

With compass, measure the fullest width of the three anterior teeth—either side—from the mesial aspect of the central incisor to the distal aspect of the cuspid.

Use this as a radius of a circle, which describe upon a piece of cardboard or thin sheet metal.

Cut out the circular disk thus secured.

With the model upon the table (teeth pointing upward), place this circular disk just inside of the edge of the six anterior teeth, the margin of the disk being on their incisive edge.

The disk thus placed always bears a definite relation to each tooth of the arch, except the third molar.

The six anterior teeth should barely show beyond the edge of the disk: these six teeth also will be found to be in an arc of a circle of 120° or one-third of a circle; the first bicuspids will show but slightly more than the cuspids; the second bicuspids will be bisected, the edge of the disk passing through the fissure of the tooth.

Usually the disk will brush the first molars somewhere on the anterior lingual third; the second molars will be entirely out of range, they continuing along in a *straight line*, for the bicuspids and molars are placed in a straight line, from the cuspids, backward, and not arched, as is often supposed. A line drawn through the sulci of these teeth will clearly demonstrate this. The *apparent* curve is due to the size, shape and contour, largely of the first and second molars. Upon connecting the distal aspect of the second molars, by means of a straight line, this line will brush along the edge of the disk when in position, thus demonstrating the equilateral form of the upper jaw and its tooth arrangement.

Now, as to the practical utility of this experiment:

Given upper and lower edentulous jaws, properly mounted on the anatomical articulator. Select teeth of proper size and shape and color; arrange them in a substantially-correct position upon the model, and wax up.

With compass, measure the fullest width of central incisor, lateral incisor and cuspid of one side, as was done on the other model. Secure the circular disk as before. Then rearrange the artificial teeth, according to the former experiment on the model of the natural teeth.

This method is an excellent and trustworthy means of securing the proper and normal arrangement of artificial teeth in edentulous jaws. It should be borne well in mind that the upward tendency of the first and second molars, corresponding with the overbite of the incisors and first bicuspid, must be carefully observed, else the Bonwill articulation cannot be secured.

If overbite be desired, and this is essential, in order to secure that scissor-like movement of the teeth, so necessary to sever and cut the food, then the sulci of the molars must be *ground deep*, and *broad*, and in the bicuspids, the sulci deepened and enlarged *mesially* and *distally*, but not directly through the center thereof. This will make possible a desirable interlocking of the cusps of the bicuspid teeth.

The upper teeth being properly placed upon the model, it is only necessary, of course, to arrange the lower teeth with regard to the uppers, and articulating well with them.—*Pacific Gazette.*

